# Curcumin Inhibits Acute Vascular Inflammation through the Activation of Heme Oxygenase-1

**DOI:** 10.1155/2018/3295807

**Published:** 2018-09-20

**Authors:** Yunjun Xiao, Junjie Xia, Shuang Wu, Ziquan Lv, Suli Huang, Haiyan Huang, Xuefen Su, Jinquan Cheng, Yuebin Ke

**Affiliations:** ^1^From the Shenzhen Key Laboratory of Molecular Epidemiology, Key Laboratory of Modern Toxicology of Shenzhen, Shenzhen Center for Disease Control and Prevention, Shenzhen, China; ^2^School of Public Health and Primary Care, Faculty of Medicine, The Chinese University of Hong Kong, Hong Kong

## Abstract

Curcumin has several therapeutic properties such as anti-inflammatory effect. Heme oxygenase-1 (HO-1) has been showed to have cytoprotective effects in some pathological conditions. However, the role of HO-1 in anti-inflammatory effect of curcumin is unknown. In this study, we investigate whether the anti-inflammatory effect of curcumin in vascular may be involved in the activation of HO-1. New Zealand white rabbits were fed regular control diet or control diet added with 0.3% curcumin (wt/wt) for four weeks. Acute vascular inflammation of rabbits was induced by putting a collar on the left common carotid artery for 24 hours. HO-1 inhibitor and siRNA were used to investigate the role of HO-1 in the anti-inflammatory effect of curcumin in collared vascular. We also explored the mechanism of curcumin-induced activation of HO-1 in vitro. The serum bilirubin and vascular, liver, and spleen HO-1 mRNA levels were significantly increased in curcumin-treated rabbits. The vascular inflammation was significantly decreased in the curcumin-treated animals compared with the control. Treatment of the rabbits with an inhibitor of HO or HO-1 siRNA to knock down the carotid artery HO-1 abolished the ability of curcumin to inhibit vascular inflammation. Treatment of cultured human artery endothelial cells with curcumin induced the HO-1 expression through the activation of nuclear factor-E2-related factor 2 (Nrf2) and an antioxidant responsive element via the p38 MAPK signalling pathway. In conclusion, curcumin inhibits vascular inflammation in vivo and in vitro through the activation of HO-1.

## 1. Introduction

Curcumin is a natural ingredient found in turmeric, which comes from the *Curcuma longa* that is commonly consumed in South and Southeast Asian countries [1]. In traditional Chinese medicine, curcumin is used to treat different diseases. Curcumin has been shown to exhibit several therapeutic properties such as anti-inflammatory, anti-oxidant, and cardiovascular protective properties [2, 3]. Studies have reported that curcumin has favourable effects in the therapy of postoperative inflammation, rheumatoid arthritis, and inflammatory bowel disease due to its anti-inflammatory properties [4–6]. The inflammation also plays an important role in the pathogenesis of vascular diseases, such as atherosclerosis and acute coronary syndrome. However, the effect of curcumin on vascular inflammation has yet to be studied.

Heme oxygenase-1 (HO-1) is one subtype of the heme oxygenases that catalyse the heme to produce iron, carbon monoxide, and biliverdin, which is converted to bilirubin by the biliverdin reductase [7]. It is well established that overexpression of HO-1 inhibits atherosclerosis, inflammation, and oxidative stress [8, 9]. Activation of HO-1 reduces atherosclerosis in LDL-receptor- and apolipoprotein E-deficient mice and prevents cardiac transplantation arteriosclerosis [10–12]. HO-1 gene delivery reduces neointima formation after vascular injury [13]. Conversely, inhibition of HO-1 exacerbates atherosclerosis [14]. HO-1-deficient mice also showed chronic inflammation [15].

Recently, it was reported that curcumin is capable of inhibiting the proliferation of vascular smooth muscle cells [16]. The antiproliferative effect of curcumin is considerably associated with its induction of HO-1. Furthermore, curcumin can activate HO-1 expression in endothelial cells [17, 18], renal epithelial cells [19], and astrocytes [20], and the activation of HO-1 in these types of cells mediates the pharmacological effects of curcumin. In this regard, the question remains as to whether the effect of curcumin on vascular inflammation is involved in the induction of HO-1. In this study, we provide evidence that curcumin inhibits acute vascular inflammation through the activation of HO-1.

## 2. Methods

### 2.1. Animals and Treatment

Forty-eight male New Zealand white (NZW) rabbits weighed approximately 2.0 kg were kept one per cage under a 12 h light/dark cycle with free access to food and water. All of the animals were fed normal rabbit diet for a week. The rabbits were then randomly divided into eight groups (*n* = 6/group) and were allowed to intake 150 g of food per day. The remaining food was weighed daily. Groups 1 and 2 were fed regular rabbit control diet. Groups 3 and 4 received the regular control diet supplemented with 0.3% (wt/wt) curcumin. The animals of groups 1 and 3 were daily intraperitoneal injected with saline. And groups 2 and 4 were daily intraperitoneal injected with the HO specific inhibitor Zinc-protoporphyrin IX (ZnPP) (7.5 mg/kg body weight) for 4 weeks.

Small interfering RNA (siRNA) transfection was used to knock down the expression of HO-1 in the remaining four groups of rabbits. These rabbits were fed regular control diet (groups 5 and 6) or control diet added with 0.3% curcumin (groups 7 and 8) for four weeks before collar implantation. The animals from groups 5 and 7 were treated with 200 *μ*L PBS containing 40 *μ*g of HO-1 siRNA (antisense: 5′-GUAGACCGGGUUCUCCUUGTT-3′, sense: 5′-CAAGGAGAACCCGGUCUACTT-3′) and 10 *μ*L FuGENE 6 (Promega) in the space between carotid artery and the collar at the time of collar implantation to knock down the expression of vascular HO-1. The animals from groups 6 and 8 were treated with 200 *μ*L PBS containing scrambled siRNA (antisense: 5′-UUCCCUCACCUGUGAGUU-AAGAACG-3′, sense: 5′-CGUUCUUAACUCACAGGU GAGGGAA-3′) in the space between carotid artery and the collar at the time of collar implantation.

All the rabbits were euthanised with sodium pentobarbital (100 mg/kg iv) 24 hours after collar implantation. The collared section of the left common carotid artery, corresponding section of the noncollared right common carotid artery, liver, and spleen were placed in ice-cold PBS and cleaned of fat and connective tissue. All procedures were approved by the Animal Care and Use Committee of the Shenzhen Centre for Disease Control and Prevention.

### 2.2. Collar Implantation

Acute vascular inflammation was induced as previously described [21]. Briefly, the rabbits were anesthetised with intravenous injection of propofol (5 mg/kg) followed by intramuscular injection of ketamine/xylazine (50/10 mg/kg). The left common carotid artery was exposed surgically and cleared of connective tissue along a 3 cm length. A nonocclusive silastic collar (length, 2 cm; internal diameter along bore, 4 mm; internal diameter at ends, 1 mm) was putted around the left common carotid artery and held in place with a nylon sleeve. The space between the collar and the artery wall was filled with PBS or siRNA and the wound was closed. 24 hours after collar implantation, the animals were sacrificed and segments from the carotid arteries were excised. The nonocclusive carotid artery collars do not affect the peak systolic velocity, lumen diameter, or shear stress in regions proximal to or within the collared segment of vessels in rabbits.

### 2.3. Immunohistochemistry

The 1 cm central portions of the noncollared and collared carotid arteries were fixed in 4% (*v*/*v*) paraformaldehyde, embedded in paraffin, and sectioned (5 *μ*m) for immunohistochemical staining. The sections were deparaffinised, and antigen retrieval was performed by incubating the slides in boiling 0.01 M citrate buffer (pH 6.0) for 10 minutes in a microwave oven at 900 W, followed by cooling at room temperature for another 30 minutes. The endogenous peroxidase was blocked by incubation in 3% (*v*/*v*) H_2_O_2_/methanol for 10 minutes. The sections were then blocked in 15% serum for 30 minutes at room temperature and incubated at 37°C for 1 hour with the mouse anti-rabbit vascular cell adhesion molecule- (VCAM-) 1 (1 : 400) and mouse anti-rabbit intercellular adhesion molecule- (ICAM-) 1 (1 : 200). Biotinylated goat anti-mouse IgG (1 : 1000) was used as a secondary antibody. Staining was visualised using the diaminobenzidine (DAB) system, followed by staining with hematoxylin. The images were digitised and analysed by an investigator blinded to the treatment of the animals. The resulting values were expressed as image units calculated by the number of pixels representing endothelial VCAM-1- and ICAM-1-positive staining divided by the circumference of the lumen.

### 2.4. Serum Bilirubin Measurement and HO Activity Assay

The serum bilirubin levels were determined using the QuantiChrom™ Bilirubin Assay Kit (Bioassay Systems). The activity of HO was assayed in the microsomes extracted from the rabbit tissues and human aortic endothelial cells (HAECs), as previously described [22]. In brief, the rabbit tissues were frozen and pulverised in liquid nitrogen, resuspended in PBS containing protease inhibitors, and then homogenised. The HAECs were lysed with three freeze thaw cycles. The tissue homogenate or cell lysate were then centrifuged at 15,000 *g* at 4°C for 20 minutes, and the supernatant was then collected and subjected to ultracentrifugation at 100,000 *g* at 4°C for 1 hour. The resulting microsomal pellet was suspended in buffer A containing 250 mmol/L sucrose and 20 mmol/L Tris (pH 7.4). The HO activity assay used a reaction mixture containing 400 to 600 *μ*g microsomal protein, 200 *μ*g rat liver microsomes, 2 mmol/L D-glucose-6-phosphate, 1 mmol/L NADPH, 1 U glucose-6-phosphate dehydrogenase, and 1 *μ*L of 2.5 mmol/L heme (Sigma) in 25% dimethyl sulfoxide (DMSO) in 100 *μ*L of buffer A on ice. The mixture was incubated at 37°C in the dark for 1 hour. The reaction was stopped by adding 100 *μ*L of ethanol/DMSO (95:5, *v*/*v*). Bilirubin was extracted following centrifugation at 13,000 *g* for 5 minutes and measured by the abovementioned assay kit. The HO activities were calculated in nanomoles of bilirubin formed per milligram of protein per hour.

### 2.5. Cell Culture

The HAECs (Cell Application Inc., San Diego, CA) were cultured in M199 medium added with foetal bovine serum (FBS) (20% *v*/*v*), streptomycin (100 *μ*g/mL), penicillin (100 U/mL), heparin (90 *μ*g/mL), and endothelial cell growth supplement (20 *μ*g/mL). The cells were cultured at 37°C 5% CO_2_ incubator and passaged 3 to 5 for the experiments.

### 2.6. Plasmids, Transfection, and Luciferase Activity Assays

To knockdown the nuclear transcription factor NF-E2-related factor (Nrf2) expression, HAECs were transfected with 200 pmol of specific Nrf2 siRNA (mixture of four different specific sequences) or a scrambled siRNA control at 37°C for 48 hours. To construct the antioxidant responsive element- (ARE-) luciferase vector, tandem repeat sequences of double-stranded oligonucleotides spanning the Nrf2 binding site 5′-TGACTCAGCA-3′ were inserted into the restriction sites of the pGL2 promoter plasmid. All of the transfection experiments were conducted using the Lipofectamine reagent kit according to the manufacturer's guidelines. For the luciferase activity assay, the cell lysate was first mixed with the luciferase substrate solution, and the luciferase activity was measured using a luminometer. For each assay, the luciferase activity was determined in three independent experiments and normalised for each sample using the *β*-galactosidase activity.

### 2.7. Total RNA Extraction and Quantitative Real-Time RT-PCR

Total RNA was isolated from the rabbit tissues and HAECs using the TRIzol reagent (Invitrogen) according to the manufacturer's instructions. cDNA was synthesised by a First-Strand cDNA Synthesis Kit. PCR assays were conducted using the ABI 7500 machine and performed in three wells for each sample. The baseline and threshold values of the amplification plots were set automatically and kept constant to obtain normalised cycle times and linear regression data. The following reaction mixture for each sample was used: 1 *μ*L cDNA, 0.8 *μ*L of primers at the final concentration of 10 μM, 10 *μ*L SYBR Green Master mix, 1 *μ*L ROX reference dye, and 7.2 *μ*L RNase-free water. For all of the experiments, the following PCR conditions were employed: denaturation at 95°C for 10 minutes, followed by 40 cycles at 95°C for 15 seconds, and then at 60°C for 1 minute. Relative levels of the mRNA expression were determined by the *Δ*ΔCT method, using the *β*-actin and 18S as controls. The PCR primers are listed in Supplemental Table 1.

### 2.8. Western Blotting

The HAECs were washed with 4°C PBS and lysed in 20 mmol/L Tris buffer (pH 7.5) containing 0.5 mmol/L EGTA-Na_2_, 0.5 mmol/L EDTA-Na_2_, and protease inhibitors. Nuclear proteins of HAECs were isolated using the EpiQuik Nuclear Extraction Kit (Epigentek). Protein concentrations were measured with a BCA kit (Pierce). Cellular proteins were detached by SDS-PAGE and electrophoretically transferred onto polyvinyl denedifluoride (PVDF) membranes. After incubation with 5% BSA in Tris-buffer saline-0.1% Tween-20 (TBST) for 1 hour at room temperature, the membranes were incubated overnight at 4°C with rabbit polyclonal antibodies against HO-1 (1 : 500) (Abbiotec), HO-2 (1 : 200) (Santa Cruz), Nrf2 (1 : 500) (Santa Cruz), p38 MAPK (1 : 1000), and phosphor-p38 MAPK (1 : 500) (Cell Signaling Technology) followed by horseradish peroxidase-conjugated secondary antibodies. Then, the membranes were exposed with a chemiluminescent substrate (Pierce). The band intensities of the membranes were quantified by the Quantity One software, and the control for equivalent protein loading was assessed with an anti-*β*-actin antibody (Sigma).

### 2.9. Statistical Analysis

The results are expressed as mean ± SEM. The Mann-Whitney *U* test or two-way ANOVA with post hoc Dunnett correction were performed to assess the differences among the groups. All of the statistical analyses were conducted by using SPSS 13.0. A probability value of less than 0.05 was considered significant.

## 3. Results

### 3.1. Curcumin Inhibits Acute Vascular Inflammation via the Activation of HO-1 in Rabbits

**Figure 1:**
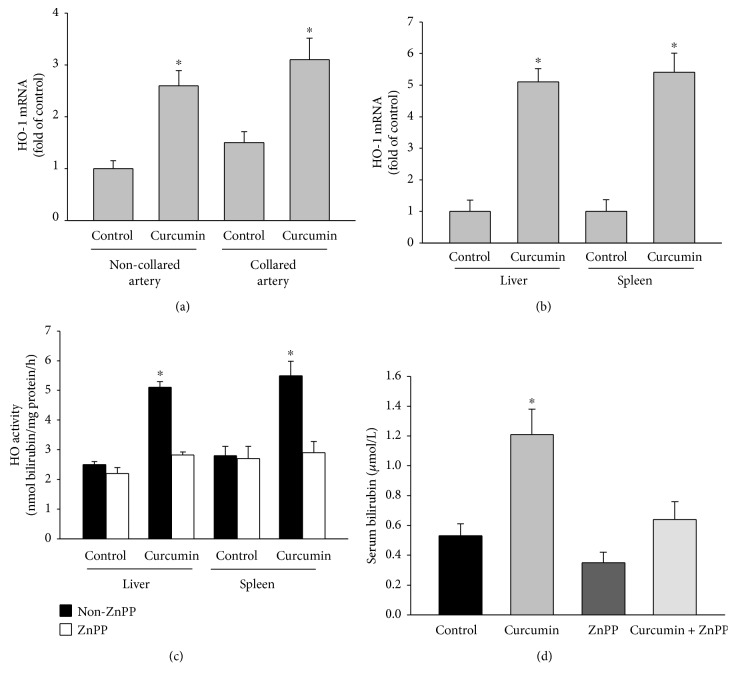
Curcumin induces HO-1 expression in NZW rabbits. (a, b) NZW rabbits were fed a control diet or control diet supplemented with 0.3% curcumin (wt/wt) for 4 weeks. During this time, the animals received daily ip injections of PBS or ZnPP (7.5 mg/kg). After four weeks, a nonocclusive silastic collar was placed around the left common carotid artery. The animals were euthanised 24 hours after collar implantation. Quantification of the HO-1 mRNA levels (fold of control) in the collared and noncollared carotid arteries (a) and in the liver and spleen (b) is shown. (c, d) HO activity in the liver and spleen (c) and serum bilirubin levels (d) from the rabbits that received the control diet or control diet supplemented with 0.3% curcumin (wt/wt) with and without ip ZnPP (7.5 mg/kg) injections. The results are represented as the mean ± SEM, *n* = 6 per group, ^∗^ indicates *P* < 0.05 compared to control.

To investigate the role of HO-1 activation in the anti-inflammatory effects of curcumin, the NZW rabbits were fed. NZW rabbits were fed regular control diet or control diet added with 0.3% curcumin (wt/wt) and daily ip injected with PBS or ZnPP for four weeks followed by carotid artery collar implantation for 24 hours. The mRNA levels of HO-1 in the collared arteries increased by 50% compared with the noncollared arteries in the control rabbits (Figure 1(a)). Curcumin supplementation elevated the mRNA levels of HO-1 in the noncollared and collared carotid arteries by 2.6 ± 0.3- and 3.1 ± 0.4-fold, respectively (Figure 1(a)). The liver and spleen mRNA levels of HO-1 were significantly increased by 5.1- and 5.5-fold in curcumin-fed animals (Figure 1(b)). Dietary supplementation with curcumin also significantly increased the liver and spleen HO activity (Figure 1(c)) and serum bilirubin levels (Figure 1(d)). The daily ip injections of ZnPP blocked the induction of HO-1 by curcumin treatment (Figures 1(c) and 1(d)).

**Figure 2:**
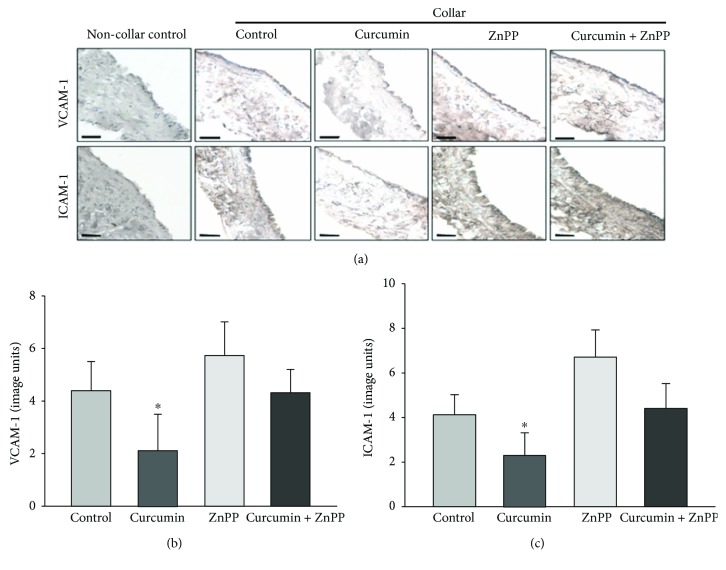
Global inhibition of HO activity by ZnPP abolishes the inhibition of acute vascular inflammation by curcumin in NZW rabbits. Acute vascular inflammation was induced by placing a silastic collar around the left carotid artery in the rabbits that received the control diet or control diet supplemented with 0.3% curcumin (wt/wt) with and without ip ZnPP (7.5 mg/kg) injections for 4 weeks. Representative sections from the collared and noncollared arteries immunostained for VCAM-1 and ICAM-1 are shown in panel (a) (bar = 50 *μ*m). Quantification of VCAM-1 and ICAM-1 is shown in (b) and (c), respectively. The results are represented as the mean ± SEM, *n* = 6 per group, ^∗^ indicates *P* < 0.05 compared to control.

Acute vascular inflammation was induced in the rabbits by placing a silastic collar around the left carotid artery for 24 hours, the carotid collar significantly increased the endothelial expression of VCAM-1 and ICAM-1 (Figure 2). Dietary supplementation with curcumin decreased the collar-induced protein expression of VCAM-1 and ICAM-1 (Figure 2). Inhibition of HO-1 expression with ZnPP injections increased vascular inflammation and significantly abolished the protective effect of curcumin on vascular inflammation.

**Figure 3:**
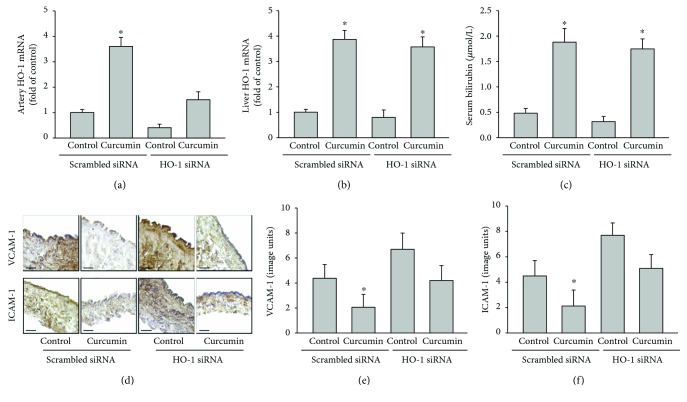
Specific inhibition of vascular HO-1 activity by HO-1 siRNA attenuates the ability of curcumin to reduce collar-induced acute vascular inflammation in NZW rabbits. NZW rabbits were fed a control diet or control diet supplemented with 0.3% curcumin (wt/wt) for 4 weeks preceding carotid artery collar implantation. HO-1 siRNA or scrambled siRNA was loaded into the space between the collar and carotid artery at the time of collar implantation. At 24 hours after collar implantation, the animals were euthanised and the carotid arteries and livers were excised for analysis. Quantification of the HO-1 mRNA levels (fold of scrambled siRNA) in the collared carotid arteries (a) and liver (b) is shown. The serum bilirubin levels are shown in (c). Representative sections from the collared arteries immunostained for VCAM-1 and ICAM-1 are shown in panel (d) (bar = 50 *μ*m). Quantification of the VCAM-1 and ICAM-1 staining in the collared artery sections is shown in (e) and (f), respectively. The results are represented as the mean ± SEM, *n* = 6 per group, ^∗^ indicates *P* < 0.05 compared to control.

We further determined the anti-inflammatory effects of curcumin by downregulating the HO-1 expression by transfection with HO-1-specific siRNA in the carotid arteries. The arterial HO-1 mRNA levels in the animals supplemented with curcumin were knocked down by 60% with HO-1 siRNA relative to the scrambled siRNA (*P* < 0.05) (Figure 3(a)). However, transfection of HO-1 siRNA in the carotid artery did not significantly reduce the curcumin-increased HO-1 expression in the liver or serum bilirubin levels (Figures 3(b) and 3(c)). Compared to the control rabbits that received scrambled siRNA, supplementation of curcumin decreased the collar-induced endothelial VCAM-1 expression by 54% and ICAM-1 expression by 57% (both *P* < 0.05) (Figures 3(d)–3(f)). Transfection with HO-1 siRNA in the collared carotid artery increased the vascular inflammation and abolished the curcumin-mediated inhibition of the collar-induced vascular inflammation (Figures 3(d)–3(f)).

To determine whether the HO-1 siRNA transfection also reduced the HO-1 activity, the thoracic aortic segments from the control rabbits were transfected with scrambled siRNA or HO-1 siRNA and then incubated with or without curcumin. The HO-1 siRNA transfection reduced the HO-1 mRNA and activity induced by curcumin (Supplemental Figure 1). Taken together, these findings suggest that the protective effect of curcumin on vascular inflammation is mediated by its induction of HO-1.

### 3.2. Curcumin Activates HO-1 in a Time- and Dose-Dependent Manner in HAECs

To assess the underlying mechanism of the activation of HO-1 by curcumin in vivo, we replicated the tests in vitro. The HAECs were treated with 100 *μ*mol/L curcumin for 1 to 24 hours or with 20 to 100 *μ*mol/L curcumin for 6 hours. The curcumin increased the HO-1 protein (Supplemental Figures 2A and 2B) and mRNA levels (Supplemental Figures 2C and 2D) in a time- and concentration-dependent manner, as measured by Western blot and quantitative RT-PCR, respectively. Furthermore, the curcumin dose dependently increased the HO activity (Supplemental Figure 2E). The induction of HO activity by curcumin was completely inhibited by ZnPP treatment (Supplemental Figure 2F). By contrast, curcumin did not significantly change the protein expression of HO-2 (Supplemental Figure 3).

### 3.3. Activation of HO-1 by Curcumin Is Mediated by Induction of Nrf2 and ARE

**Figure 4:**
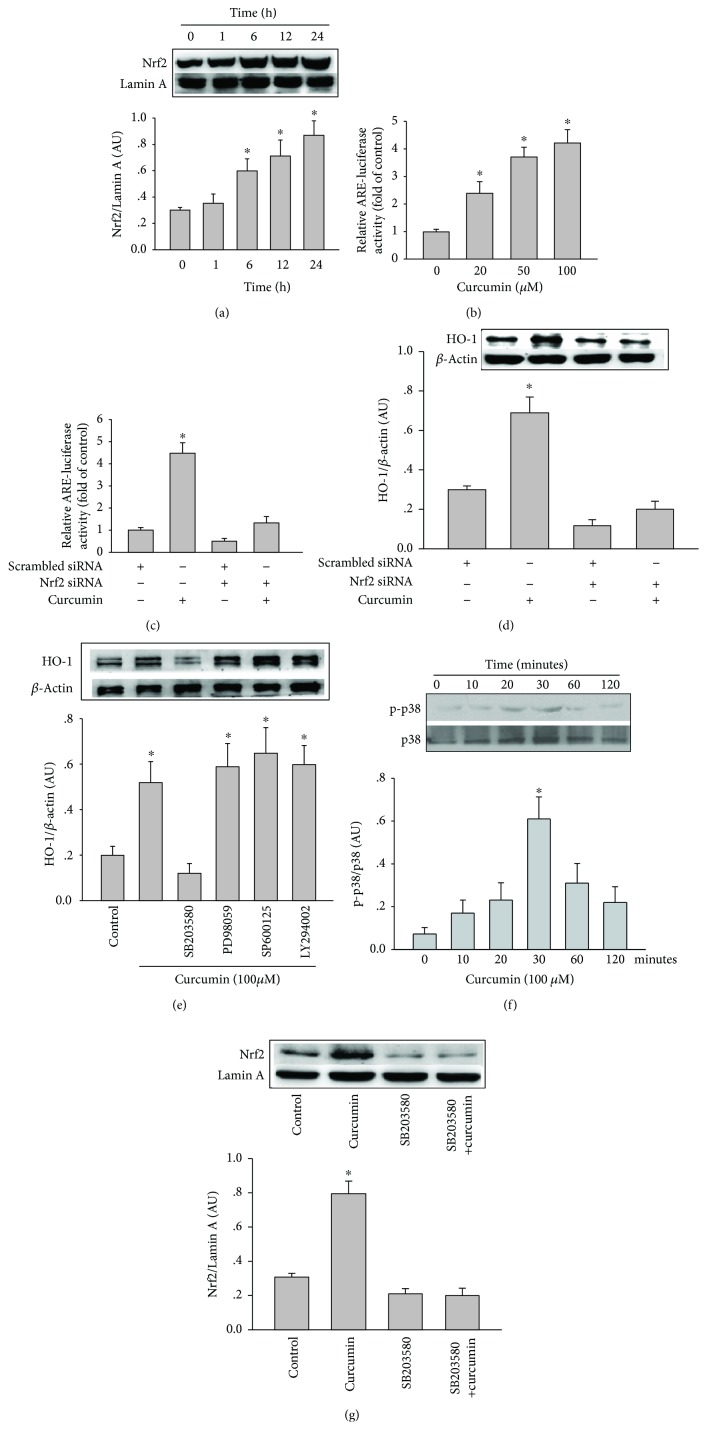
Curcumin induces HO-1 expression and ARE activation in HAECs by activating Nrf2 and the p38-MAPK signalling pathway. (a) HAECs were incubated with 100 μmol/L curcumin for the indicated times. The Nrf2 protein expression in the nuclear extracts was analysed by Western blotting. The results are quantified as the Nrf2 intensity relative to Lamin A. (b) Ten hours after transfection with an ARE luciferase or control vector, HAECs were incubated with the indicated concentrations of curcumin for 6 hours. Cell lysates were assayed for luciferase activity as the fold induction by normalising the transfection efficiency and dividing the values of each experiment relative to the control. The ARE-luciferase activity (c) and HO-1 protein expression (d) were analysed by luciferase assay and Western blotting and, respectively, in lysates of HAECs transfected with scrambled siRNA or Nrf2 siRNA for 48 hours, then incubated with or without 100 *μ*mol/L curcumin for a further 6 hours. The results are quantified as the HO-1 intensity relative to *β*-actin or the luciferase activity fold induction relative to the control. (e) HO-1 protein expression was analysed by Western blotting in lysates of HAECs preincubated with 10 *μ*mol/L SB203580, PD098059, SP600125, or LY294002 for 1 hour and then incubated with or without 100 *μ*mol/L curcumin for a further 6 hours. (f) Phosphorylated p38-MAPK and p38-MAPK were analysed by Western blotting in lysates of HAECs incubated with 100 *μ*mol/L curcumin for the indicated times. The results are quantified as the phosphorylated p38-MAPK relative to the total p38. (g) The Nrf2 protein expression in the nuclear extracts was analysed by Western blotting in lysates of HAECs incubated with 100 *μ*mol/L curcumin with or without p38-MAPK inhibitor SB203580. The results are quantified as the Nrf2 intensity relative to Lamin A. All data are expressed as the mean ± SEM of three independent experiments.^∗^ indicates *P* < 0.05 compared to cells incubated without control.

The exposure of cells to natural antioxidants possessing Michael-reaction acceptors dissociated the Kelch-like ECH-associated protein 1 (Keap1)-Nrf2 complex, promoting the translocation of Nrf2 into the nucleus, where it binds to ARE and activates the gene transcription of HO-1 [19]. To investigate whether curcumin promotes the translocation of Nrf2 into the nucleus, Western blot assay of nuclear protein of curcumin-treated HAECs found a gradual increase in Nrf2 levels with time (Figure 4(a)). HAECs transfected with ARE luciferase plasmid were treated with curcumin, and the luciferase activity of ARE were measured. Curcumin also concentration dependently elevated the luciferase activity of ARE (Figure 4(b)). The role of Nrf2 in the curcumin-induced HO-1 and ARE activation was assessed by transfecting with Nrf2 siRNA or scrambled siRNA in HAECs and then treating with curcumin. Transfection of Nrf2 siRNA reduced the expression of HO-1 and ARE luciferase activity activated by curcumin (Figures 4(c) and 4(d)), suggesting that curcumin-induced activation of HO-1 and ARE is mediated by the nuclear translocation of Nrf2.

### 3.4. Curcumin Activates the p38-MAPK Signalling Pathway and Induces Nrf2 Activation and HO-1 Expression

The binding of Nrf2 to Keap1 is regulated by several signalling pathways such as c-Jun N-terminal kinase (JNK), extracellular signal-regulated kinase (ERK), and p38 MAPK signalling pathways [23]. To investigate the role of the individual MAPK and PI3K/Akt signalling pathways in the curcumin-induced HO-1, we assessed the effects of SB203580, PD098059, SP600125, and LY294002, which are specific inhibitors of the p38 MAPK, ERK, JNK, and PI3K/Akt signalling pathways, respectively, on the HO-1 protein expression. Treatment of the HAECs with SB203580 reduced the curcumin-induced HO-1 expression by 56% (Figure 4(e)). In contrast, PD098059, SP600125, and LY294002 had no effect on the curcumin-increased HO-1 expression. When the HAECs were incubated with 100 *μ*mol/L curcumin for 10 to 120 minutes, p38-MAPK was rapidly phosphorylated, with maximal phosphorylated-p38 protein expression occurring after 30 minutes (*P* < 0.05) (Figure 4(f)). Pretreatment of HAECs with SB203580 significantly abolished curcumin-induced Nrf2 activation (Figure 4(g)). These results suggest that p38 MAPK mediated the curcumin-induced Nrf2 activation and HO-1 expression in HAECs.

### 3.5. Curcumin Inhibits TNF-*α*-Stimulated Inflammation by Activating HO-1 in HAECs

**Figure 5:**
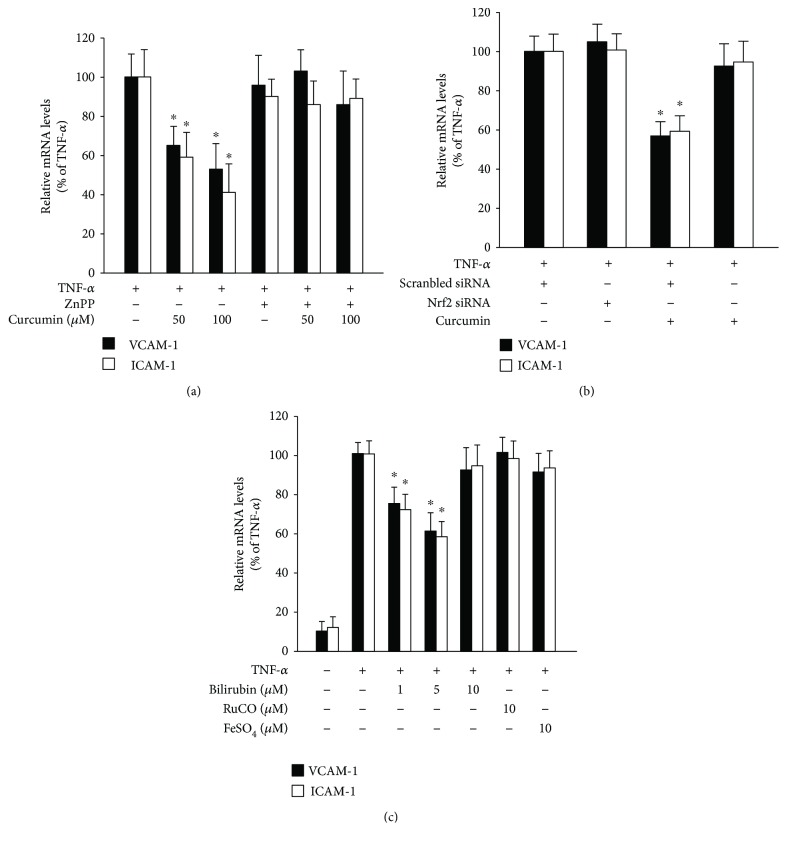
Curcumin and bilirubin inhibit TNF-*α*-induced inflammation in HAECs via the induction of HO-1 expression. (a) The VCAM-1 and ICAM-1 mRNA levels were quantified by real-time RT-PCR in HAECs preincubated with the indicated concentrations of curcumin for 16 hours in the absence or presence of ZnPP (final concentration 20 *μ*mol/L) and then stimulated with TNF-*α* (1 ng/mL) for a further 6 hours. (b) The VCAM-1 and ICAM-1 mRNA levels in HAECs transfected with scrambled siRNA or Nrf2 siRNA for 48 hours, incubated with or without 100 *μ*mol/L curcumin for 16 hours, and then stimulated with TNF-*α* (1 ng/mL) for a further 6 hours. (c) The VCAM-1 and ICAM-1 mRNA levels in HAECs preincubated with bilirubin, RuCO, or FeSO_4_ at the indicated concentrations for 2 hours and then stimulated with TNF-*α* (1 ng/mL) for a further 6 hours. All data are expressed as the mean ± SEM of three independent experiments. ^∗^ *P* < 0.05 compared with cells stimulated with TNF-*α* without curcumin or bilirubin.

Next, to examine whether the activation of HO-1 by curcumin inhibits TNF-*α*-stimulated inflammation in vitro, HAECs were treated with curcumin and ZnPP before TNF-*α* treatment. Treatment with 50 and 100 *μ*mol/L curcumin decreased the TNF-α-induced increase in the mRNA levels of VCAM-1 and ICAM-1, which was blocked by treatment with ZnPP (Figure 5(a)). We further determined whether the anti-inflammatory effect of curcumin in TNF-*α*-activated HAECs also involved Nrf2 activation. When the HAECs were treated with Nrf2 siRNA, curcumin did not reduce the TNF-*α*-induced increase in mRNA levels of VCAM-1 and ICAM-1 (Figure 5(b)). These findings suggest that curcumin activates HO-1 through the induction of Nrf2, which then inhibits the TNF-*α*-stimulated inflammation in HAECs. Because curcumin elevated the serum bilirubin concentration in rabbits, we further determined whether bilirubin has anti-inflammatory effects in vitro. HAECs were incubated with 1 to 10 *μ*mol/L bilirubin preceding TNF-*α* stimulation. The bilirubin dose-dependently reduced the TNF-*α*-stimulated mRNA levels of VCAM-1 and ICAM-1 (Figure 5(c)). In contrast, the CO and Fe^2+^ other two enzymatic products of HO-1 did not inhibit the TNF-*α*-stimulated inflammation. These findings indicate that the protective effects of curcumin against vascular inflammation in rabbits may be mediated by the elevated serum bilirubin concentration.

## 4. Discussion

Curcumin has been shown to have several cellular properties such as antioxidant, anti-inflammatory, antiproliferative, proapoptotic, and anticancer effects [24]. However, whether curcumin can protect against vascular inflammation and the corresponding molecular mechanisms through which it may achieve this have not been clearly elucidated. This study provides evidence that curcumin inhibits vascular inflammation via the induction of HO-1 expression in vivo and in vitro. We further show that the induction of HO-1 by curcumin involves nuclear translocation of Nrf2 and ARE activation via the p38-MAPK signalling pathway.

An extensive body of research has shown that curcumin can exert anti-inflammatory effects through different mechanisms, e.g., curcumin inhibits the nuclear factor-*κ*B (NF-*κ*B) activation, which may participate in various stimuli in steatohepatitis mice, intestine epithelial cells, and microglial cells [25, 26]. Moreover, curcumin can inhibit the independent MAPK pathways, which are activated by most of the inflammatory stimuli [27]. In this study, we found that another mechanism of the anti-inflammatory effects of curcumin involves the activation of HO-1. Curcumin has been shown to activate the HO-1 and exerts different cytoprotective properties in different cells. For example, curcumin can activate HO-1 expression and inhibit the proliferation of vascular smooth muscle cells [16]. In bovine aortic endothelial cells, curcumin can protect against oxidative stress by increasing the HO activity [17]. Moreover, curcumin has been shown to increase the HO-1 mRNA expression and inhibit inflammation in the lungs of lipopolysaccharide-treated mice and carrageenan-induced acute inflammation in rats [28, 29]. We extend these findings by showing that dietary supplementation with curcumin inhibited collar-induced acute vascular inflammation in NZW rabbits by increasing the HO-1 expression and serum bilirubin levels, which were blocked by injections with the HO specific inhibitor ZnPP.

In contrast to the abolish of the anti-inflammatory effects of curcumin in rabbits with complete inhibition of HO by ZnPP, knockdown of vascular HO-1 by specific siRNA only partially blocked the inhibition of curcumin on collar-induced acute vascular inflammation (Figure 3(f)). This may be explained by the low- and short-time siRNA transduction in the vascular tissue. Moreover, localised vascular knockdown of HO-1 did not completely block the curcumin-mediated increase in the liver HO-1 mRNA expression and serum bilirubin levels (Figures 3(b) and 3(c)). This result suggests that curcumin induces HO-1 expression in different tissues including the arteries, spleen, and liver and elevates the circulating concentration of bilirubin the metabolic product of HO-1. In addition, treatment of HAECs with bilirubin protected against TNF-*α*-induced endothelial inflammation (Figure 5(c)). This suggests that the curcumin-induced increase in the serum bilirubin concentration as a result of the activation of HO-1 expression may have mediated the inhibition of curcumin on acute vascular inflammation in the collar-treated rabbits. Thus, the serum bilirubin concentration remained increased in the curcumin-treated rabbits with specific inhibition of artery HO-1, which may explain the discrepancy between the anti-inflammatory effects of curcumin in rabbits with complete block of HO activity by ZnPP and local knockdown of artery HO-1 by HO-1-specific siRNA.

The nuclear transcriptional factor Nrf2 has recently been considered as an important regulator in the induction of ARE-response gene expression. Nrf2 resides in the cytosol, where it binds to the inhibitor Keap1. Under different stimulation, Nrf2 is dissociated from Keap1 and translocates into the nucleus, where it binds to the ARE in the promoter region of target genes such as HO-1 [30]. The binding of the translocated Nrf2 to the multiple copies of ARE in the promoter region of the HO-1 increases the HO-1 expression [31]. It has been shown that curcumin dissociates the Nrf2-Keap1 complex, resulting in an increased binding of Nrf2 to ARE, which mediates the transcriptional activation of HO-1 [19]. Our data show that curcumin increases the translocation of Nrf2 into the nucleus and increases the ARE activity and finally activates the expression of HO-1, which are abolished by transfection with Nrf2 siRNA in cultured HAECs (Figures 4(c) and 4(d)). This indicates that the curcumin-activated HO-1 expression is mainly mediated by ARE activation via the Nrf2 transcriptional regulation.

Studies have suggested that different signalling pathways participate in Nrf2 translocation and HO-1 activation [23]. MAPKs including p38 MAPK, JNK, and ERK have important roles in Nrf2 translocation and activate the HO-1 expression in different cells [9]. Some studies have reported that curcumin induces HO-1 activation through the signalling pathways of PKC*δ* and p38 MAPK in hepatocytes and renal epithelial cells [19, 32, 33]. Curcumin also inhibits the cytokine secretion within LPS-stimulated monocytes through the induction of HO-1 via the activation of PKC*α*, PKC*δ*/ERK1/2, p38, and PI3-kinase [28]. In addition, bisdemethoxycurcumin, an analogue of curcumin, inhibits LPS-stimulated inflammation in macrophages through the activation of HO-1 expression via a Ca^2+^/calmodulin-dependent protein kinase II–ERK1/2-Nrf2 cascade [34]. Moreover, demethoxy curcuminoids regulate HO-1 expression via Nrf2 activation through a PI3K/Akt signalling pathway in mouse *β*-cells [35]. In this study, treatment of HAECs with curcumin rapidly increased the p38 MAPK phosphorylation, which has been shown for inflammatory stimuli such as interleukin-10 [8] and other stimuli [36]. However, although curcumin regulates the MAPK signalling pathways in several different cells, the findings are somewhat inconsistent. Under some conditions, curcumin inhibits the activation of MAPKs. For example, curcumin inhibits the p38 MAPK phosphorylation upon to the stimulation of vascular endothelial growth factor (VEGF) in human intestinal microvascular endothelial cells [37]. This antiangiogenic effect of curcumin has potential clinical benefits in treating gut inflammation and tumour. Other studies paradoxically reported that curcumin activated the MAPK signalling pathways such as JNK in human colon cancer HCT116 cells [38] and p38 MAPK in human neutrophils [39]. Our findings demonstrate that the selectivity inhibition of p38 MAPK signalling pathway, but not JNK or ERK, abolishes HO-1 activation by curcumin in HAECs. The discrete mechanism of the early phosphorylation of p38 MAPK that induces the HO-1 transcription activation by curcumin in HAECs needs to be further studied.

In summary, we have found that curcumin protects against acute vascular inflammation through the activation of HO-1 by the nuclear translocation of Nrf2 and ARE activation via the p38 MAPK signalling pathway. These results provide new insights into the mechanisms of the anti-inflammatory effects of curcumin and indicate that the inhibition of vascular inflammation by curcumin-induced HO-1 activation may be useful for much of the vascular damage that occurs as a result of acute coronary events.

## Data Availability

The data used to support the findings of this study are available from the corresponding author upon request.

## Abbreviations

ARE: Antioxidant responsive element
HAECs: Human aortic endothelial cells
HO-1: Heme oxygenase-1
ICAM-1: Intercellular adhesion molecule-1
Nrf2: Nuclear factor-E2-related factor 2
VCAM-1: Vascular cell adhesion molecule-1
ZnPP: Zinc-protoporphyrin IX.

## Supplementary Materials

Supplementary Materials Supplemental Table 1: PCR primer sequences. Supplemental Figure 1: effects of HO-1 siRNA on the curcumin-mediated induction of HO-1 mRNA levels and HO activity in the thoracic aorta. Supplemental Figure 2: induction of HO-1 expression by curcumin in HAECs. Supplemental Figure 3: curcumin does not affect the HO-2 protein expression in HAECs.
