# Supplementary material for: Curcumin Inhibits Acute Vascular Inflammation through the Activation of Heme Oxygenase-1
Source: Oxid Med Cell Longev. 2018 Sep 20;2018:3295807. doi: 10.1155/2018/3295807 (PMC6171216; doi:10.1155/2018/3295807)
Supplement: Supplementary Materials — Supplemental Table 1: PCR primer sequences. Supplemental Figure 1: effects of HO-1 siRNA on the curcumin-mediated induction of HO-1 mRNA levels and HO activity in the thoracic aorta. Supplemental Figure 2: induction of HO-1 expression by curcumin in HAECs. Supplemental Figure 3: curcumin does not affect the HO-2 protein expression in HAECs. [file 3295807.f1.doc]

**Supplemental Table 1.** PCR primer sequences

| Gene | Forward sequence | Reverse sequence |
| --- | --- | --- |
| Rabbit β-actin | GAT CGC TGA CCG TAT G | GTC GTA CTC CTG CTT GGT G |
| Rabbit 18S | CGG CTA CCA CAT CCA AGG AA | GCT GGA ATT ACC GCG GCT |
| Rabbit HO-1 | TGG AGC TGG ACA TGG CCT TC | TCT GGG CGA TCT TCT TAA GG |
| Human HO-1 | TTG CTG TAG GGC TTT ATG C | CTG CAT TTG AGG CTG AGC C |
| Human VCAM-1 | ATG TAG TGT CAT GGG CTG TG | GGA ATG AGT AGA GCT CCA CC |
| Human ICAM-1 | CCA TCT ACA GCT TTC CGG CGC | CTC TGG GGT GGC CTT CAG CA |
| Human 18S | AGT CCC TGC CCT TTG TAC ACA | GAT CCG AGG GCC TCA CTA AAC |
| Human β-actin | GAT CGC TGA CCG TAT GCA G | GTC GTA CTC CTG CTT GGT G |


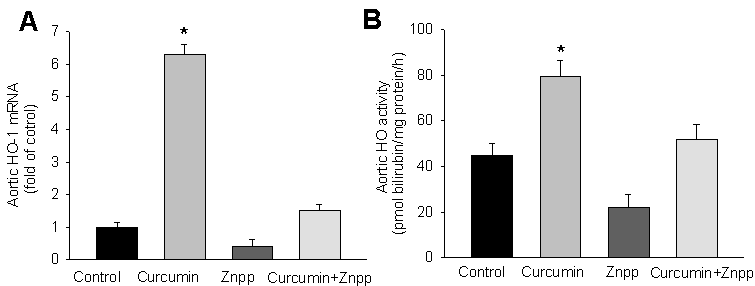


**Supplemental Figure 1.** Effects of HO-1 siRNA on the curcumin-mediated induction of HO-1 mRNA levels and HO activity in the thoracic aorta. Thoracic aortic segments isolated from rabbits that had received the control chow were incubated at 37°C in a humidified 5% CO_2_ incubator with an endothelial cell growth medium-serum free culture medium. The segments were transfected by incubation at 37°C for 48 hours with 400 pmol of HO-1 siRNA or scrambled siRNA, and then incubated with or without 100 μmol/L curcumin for a further 6 hours. The HO-1 mRNA levels were quantified by real-time PCR and expressed as the fold change relative to scrambled siRNA (**A**). The HO activity is shown in **B.** Data are expressed as the mean ± SEM of three independent experiments. ^*^ indicates*P*<0.05 compared to control.

.

**
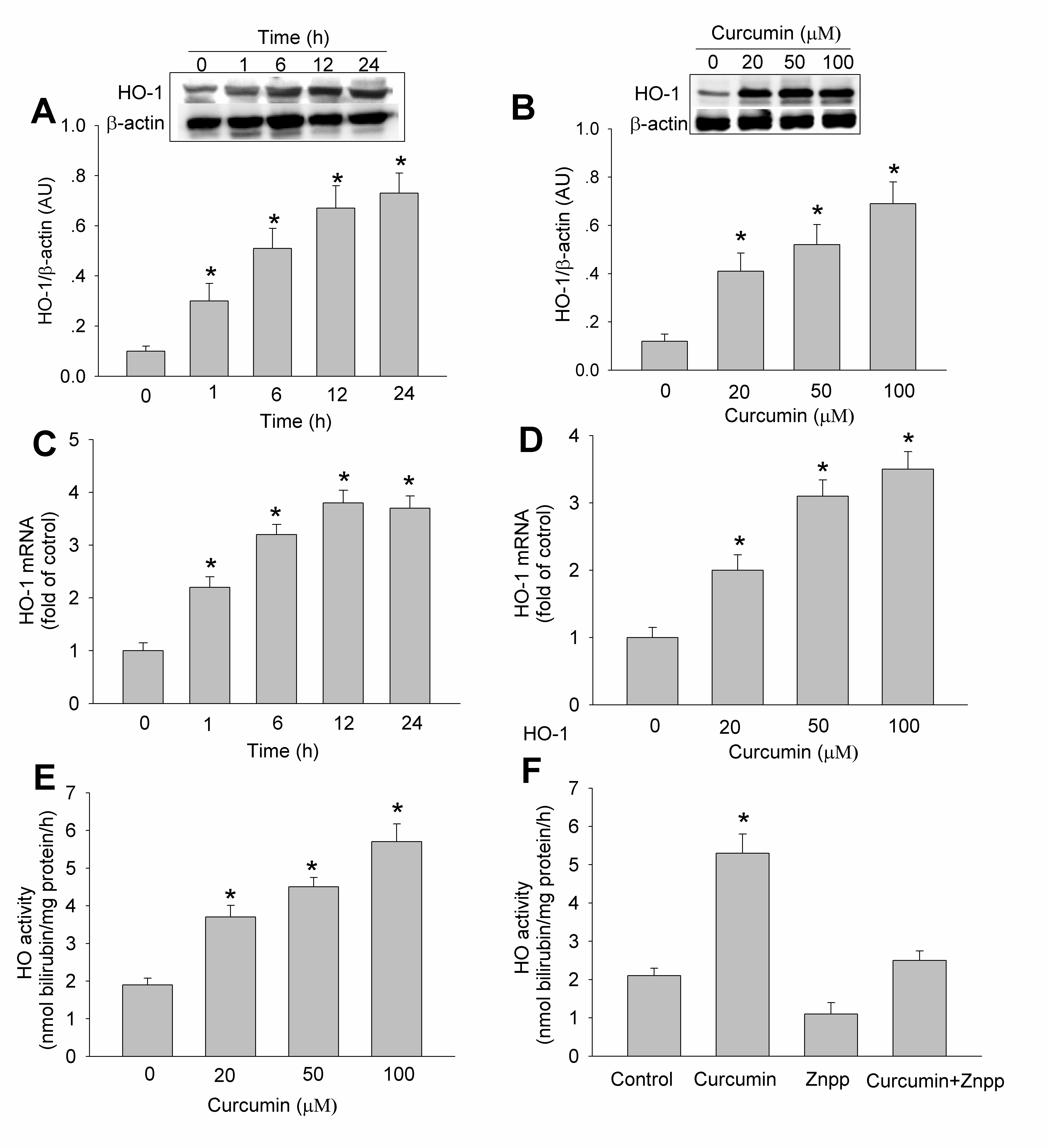
**

**Supplemental Figure 2.** Induction of HO-1 expression by curcumin in HAECs. **A,** Time-dependent induction of HO-1 protein expression by curcumin. Cultured HAECs were incubated with 100 μmol/L curcumin for the indicated times. **B,** Dose-dependent induction of HO-1 protein expression by curcumin. HAECs were treated with curcumin for 6 hours at the indicated concentrations. Cell lysates were subjected to SDS-PAGE and Western blotting with anti-HO-1 or anti-β-actin antibodies as indicated. The results represent the intensity of the HO-1 band relative to β-actin. The relative HO-1 mRNA expressions (fold of control) quantified by real-time RT-PCR are shown in **C** and **D. E,** Dose**-**dependent induction of HO-1 activity by curcumin. **F,** HO-1 activity in HAECs incubated with 100 μmol/L curcumin for 6 hours in the absence or presence of ZnPP (final concentration 20 μmol/L). All data are expressed as the mean ± SEM of three independent experiments. ^*^ indicates*P*<0.05 compared to control.


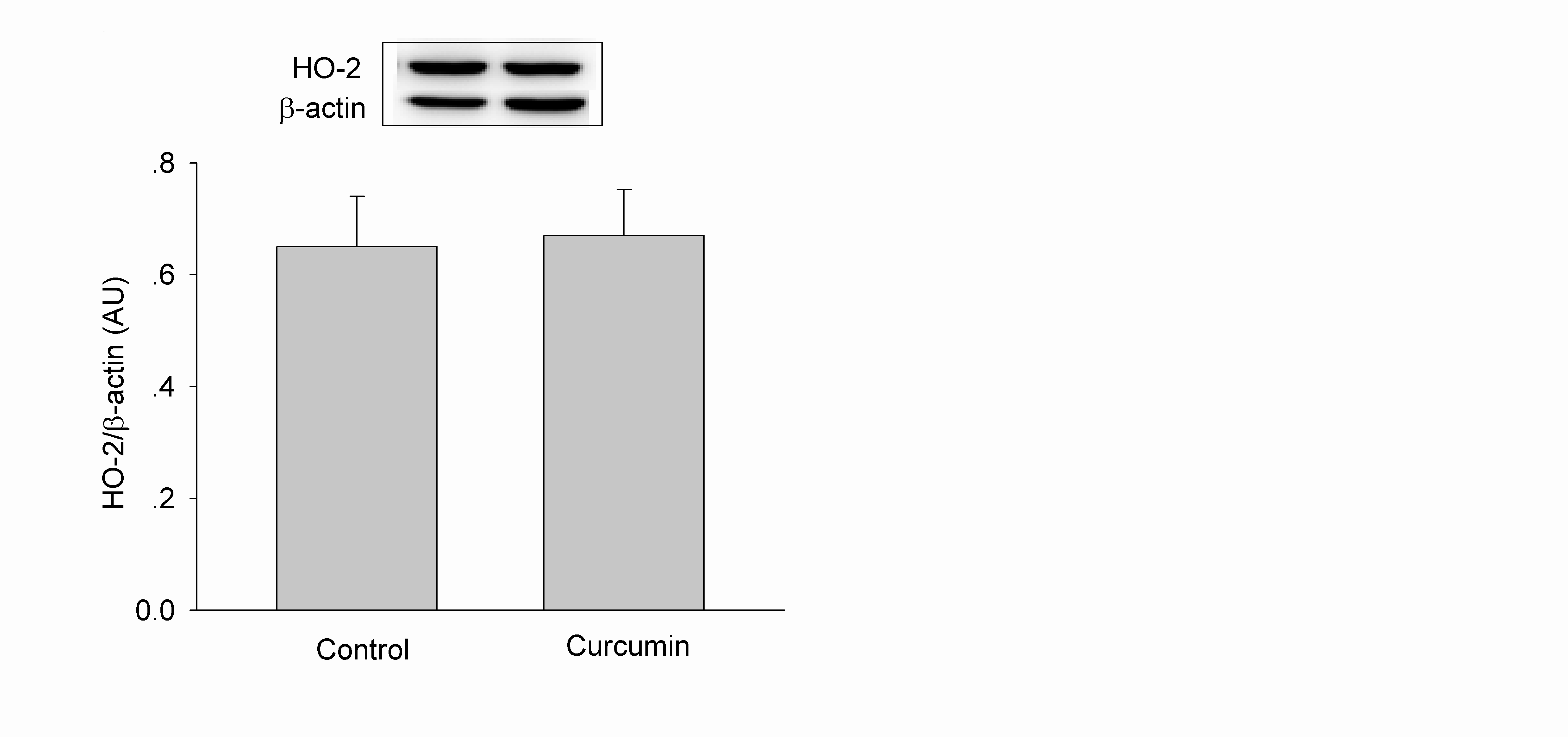


**Supplemental Figure 3.** Curcumin does not affect the HO-2 protein expression in HAECs. HAECs were incubated with or without 100 μmol/L curcumin for 6 hours, and the cell lysates were subjected to SDS-PAGE and Western blotting with anti-HO-2 or anti-β-actin antibodies as indicated. The results represent the intensity of the HO-1 band relative to β-actin. Data are expressed as the mean ± SEM of three independent experiments.
